# Molecular Characterization of a Debilitation-Associated Partitivirus Infecting the Pathogenic Fungus *Aspergillus flavus*

**DOI:** 10.3389/fmicb.2019.00626

**Published:** 2019-03-28

**Authors:** Yinhui Jiang, Jingxian Wang, Bi Yang, Qinrong Wang, Jianjiang Zhou, Wenfeng Yu

**Affiliations:** ^1^Key Laboratory of Endemic and Ethnic Diseases, Guizhou Medical University, Ministry of Education, Guiyang, China; ^2^Key Laboratory of Medical Molecular Biology, Guizhou Medical University, Guiyang, China; ^3^Experiment Center of Stem Cell and Tissue Engineering Research, Guizhou Medical University, Guiyang, China

**Keywords:** *Aspergillus flavus*, dsRNA mycovirus, *Aspergillus flavus* partitivirus 1, unclassified group, debilitation symptoms, murine pathogenicity

## Abstract

The opportunistic human pathogenic fungus *Aspergillus flavus* is known to be infected with mycoviruses. In this study, we report a novel mycovirus *A. flavus* partitivirus 1 (AfPV1) that was originally isolated from the abnormal colonial morphology isolate LD-3-8 of *A. flavus*. AfPV1 has spherical virus-like particles about 40 nm in diameter, and three double-stranded RNA (dsRNA) segments (dsRNA1, 2, and 3 with lengths of 1.7, 1.4, and 1.1 kbp, respectively) were packaged in the virions. dsRNA1, dsRNA2, and dsRNA3 each contained a single open reading frame and potentially encoded 62, 42, and 32 kDa proteins, respectively. The dsRNA1 encoded protein shows similarity to the RNA-dependent RNA polymerase (RdRp) of partitiviruses, and the dsRNA2 product has no significant similarity to any other capsid protein (CP) in the GenBank databases, beside some homology with the hypothetical “capsid” protein of a few partitiviruses. The dsRNA3 encodes a protein with no similarity to any protein in the GenBank database. SDS-PAGE and polypeptide mass fingerprint-mass spectrum (PMF-MS) analyses indicated that the CP of the AfPV1 was encoded by dsRNA2. Phylogenetic analysis showed that the AfPV1 and relative viruses were found in an unclassified group inside the *Partitiviridae* family. AfPV1 seems to result in debilitation symptoms, but had no significant effects to murine pathogenicity. These findings provide new insights into the partitiviruses taxonomy and the interactions between viruses and *A. flavus*.

## Introduction

Mycoviruses are widespread in almost all major groups of fungi (Ghabrial and Suzuki, 2009; Pearson et al., 2009), and most cause no obvious effects in their hosts, but some do cause obvious symptoms resulting in debilitated virulence, slow growth rate, and poor sporulation (Ghabrial and Suzuki, 2009; Yu et al., 2010; Liu et al., 2014; Xie and Jiang, 2014). Currently recognized mycoviruses have single-stranded (ss) or double-stranded (ds) RNA, or rarely DNA, as their genetic material (Yu et al., 2010, 2013; Liu et al., 2014). Members of family *Partitiviridae* are classified into four genera, namely *Alphapartitivirus,* *Betapartitivirus,* *Gammapartitivirus,* and *Deltapartitivirus* (Vainio et al., 2018). Partitiviruses generally possess two essential dsRNA genome segments (1.3–2.5 kbp in length) containing an open reading frame (ORF), respectively (Nibert et al., 2014). The larger one encodes an RNA dependent RNA polymerase (RdRp) and the smaller one encodes the capsid protein (CP) (Nibert et al., 2014). Partitiviruses are generally encapsidated in isometric particles about 25–40 nm in diameter. Recently, partitivirus capsids were shown to have a so-called “*T* = 2” organization comprising 60 CP dimers arranged on a *T* = 1 icosahedral lattice, as determined by cryo-electron microscopy and three-dimensional (3D) image reconstruction, as well as one determined by X-ray crystallography (Ochoa et al., 2008; Pan et al., 2009; Tang et al., 2010; Xiao et al., 2014). To date, most partitiviruses are typically associated with no obvious effects on their fugal hosts (Nibert et al., 2014). Recently, some interesting reports have clarified about the role of partitivirus in resistance to salinity, regulation of mycotoxin production, and for affecting biocontrol potential in their hosts (Nerva et al., 2017, 2018; Chun et al., 2018).

*Aspergillus flavus* is an opportunistic pathogen causing aspergillosis diseases in the immunocompromised human population, and the fungus is the second-leading pathogen causing invasive and non-invasive aspergillosis, next to *Aspergillus fumigatus* (Krishnan et al., 2009; Amaike and Keller, 2011). In addition, *A. flavus* produces secondary metabolite aflatoxin, the most toxic, and potent hepatocarcinogenic natural compound (Klich, 2007). Until recent years, the only drugs available to treat aspergillosis are amphotericin B, itraconazole, voriconazole, posaconazole, and caspofungin (Hedayati et al., 2007). But *A. flavus* infections are often complicated by resistance or refractoriness to current antimicrobial agents (Arendrup, 2014; Paul et al., 2015). Therefore, an urgent need exists for new therapeutic strategies to control *A. flavus* infections.

With the increasing discovery of mycoviruses that are able to selectively infect human pathogenic fungi, mycoviruses have shown the significant potential for biological control agents (Van De Sande et al., 2010; Bhatti et al., 2011; Özkan and Coutts, 2015). So it seems possible that some mycovirus conferring hypovirulence could be used as virus control agents for *A. flavus* infection. Mycoviruses in *A. flavus* have been detected, but usually remain latent and seldom induce symptoms (van Diepeningen et al., 2008). Mycoviruses that confer hypovirulence are generally characterized by a reduction in conidiation, pigmentation, and growth rate (Son et al., 2015). In order to obtain the mycoviruses that cause obvious symptoms in *A. flavus*, 417 *A. flavus* isolates collected from patients and patient’s room, were tested for mycovirus elements and morphological observation in this study. At last, a novel partitivirus, *A. flavus* partitivirus 1 (AfPV1), causing obvious symptoms on its host morphology was found. The influence of AfPV1 on pathogenicity of *A. flavus* generating virus-infected and virus-free isogenic lines and the unique characteristics of AfPV1 were analyzed and discussed here.

## Materials and Methods

### Fungal Isolates and Growth Conditions

The single-spore isolate *A. flavus* LD-3-8 was isolated from patient’s room of Affiliated Hospital of Guizhou Medical University. A virus-free isolate LD-F was obtained from isolate LD-3-8 by single asexual spore isolates. Isolate LD-F1 derived from LD-F, was labeled with a pyrithiamine resistance (*ptr*) gene using PEG-mediated methods. All *A. flavus* isolates were cultured on potato dextrose agar (PDA) plates and Czapek Agar (CZ) plate at 30°C and stored on PDA slants at 4°C.

### Extraction and Purification of dsRNA From Mycelia

Mycelial agar plugs of each isolate of *A. flavus* were cultured on sterilized cellophane films placed on PDA plates (9 cm in diameter) at 30°C in the dark for 3–5 days. Mycelia in each plate were harvested and stored at -80°C until use. Extraction and purification of the dsRNAs from mycelia was carried out using CF-11 cellulose (Sigma, St. Louis, MO, United States) column chromatography as previously described (Morris and Dodds, 1979). DsRNA in each extract was analyzed by electrophoresis on an agarose gel (1%, wt/vol), stained with ethidium bromide and viewed on an UV *trans*-illuminator.

### cDNA Cloning, Sequencing, and Sequence Analysis

The dsRNA elements (1.7 kb for dsRNA1, 1.4 kb for dsRNA2, and 1.1 kb for dsRNA3) extracted from isolate LD-3-8 of *A. flavus* were separated by agarose gel electrophoresis, and the individual dsRNA bands were cut off for further analysis. cDNA synthesis and molecular cloning of these dsRNAs were performed using the random-primer (5′-CGATCGATCATGATGCAATGCNNNNNN-3′) amplification method, sequencing of the cDNAs, and analysis of the sequences were done using the procedures described in our previous studies (Jiang et al., 2015). The terminal sequences of each dsRNA were cloned using the modified method described previously (Potgieter et al., 2009).

The full-length cDNA sequence for dsRNA1, dsRNA2, or dsRNA3 was obtained from assembling the partial sequences in the different cDNA clones. ORFs in full-length cDNA sequence were searched using DNAMAN 7.0 (Lynnon Biosoft, Quebec, Canada). Amino acid sequences were aligned using Clustal W implemented in MEGA 6.06 (Tamura et al., 2013). The phylogenetic tree was constructed using the Maximum Likelihood method with a Jones-Taylor-Thornton (JTT) with freqs model, and the optimal trees were statistically evaluated with a bootstrap of 1,000-replicates using MEGA 6.06 (Tamura et al., 2013).

### Purification, Observation, and Polypeptide Mass Fingerprint-Mass Spectrum (PMF-MS) Analyses of Virus Particles

The viral particles (VLPs) were isolated from *A. flavus* isolate LD-3-8 using the modified method described previously (Yu et al., 2010). The isolates were cultured at 30°C for 5 days on sterilized cellophane films placed on PDA. Approximately 25 g mycelia were ground in liquid nitrogen and mixed with four volumes of 0.01 M phosphate buffer saline (PBS) (pH 7.4), and then the mixture was gently shaken on ice for 30 min. The mixture was separated with high-speed centrifugation (10,000 ×*g* for 30 min). The supernatant was then subjected to ultracentrifugation at 120,000 × *g* for 2 h under 4°C to precipitate the virus particles in the supernatant. The supernatant containing the virus particles was then overlaid on a centrifuge tube containing sucrose density gradient (10–40%) and centrifuged at 70,000 ×*g* for 3 h at 4°C. The gradient containing VLPs was diluted with PBS and ultracentrifuged at 120,000 ×*g* for 2 h at 4°C. Five separated fractions were individually measured for the presence of the virus particles by dsRNA detection using the agarose gel electrophoresis. The fractions containing virus particles were carefully collected and suspended using 200 μl PBS. The virus particles were observed under transmission electron microscopy (TEM) after staining with 2% (wt/vol) phosphotungstic acid solution (pH 7.4).

The purified virus particles suspension was loaded on a sodium dodecyl sulfate (SDS)-polyacrylamide (12%) gel stained with Coomassie brilliant blue R250. The resulting protein bands on the gel were individually excised and subjected to matrix-assisted laser desorption/ionization-time of flight mass spectrometry (MALDI-TOF-MS) analysis at Sangon Biotech (Shanghai, China). To avoid missing any proteins during virion preparation, purified virus particles suspension was also directly subjected to liquid chromatography-tandem mass spectrometry (LC-MS/MS) analysis at Sangon Biotech (Shanghai, China).

### Horizontal Transmission of Mycoviruses AfPV1

A virus-free isolate LD-F was obtained from isolate LD-3-8 by single asexual spore isolates, and then was labeled with a *ptr* gene using PEG-mediated methods as previously described (Chang et al., 2010). The *ptr* isolate (named LD-F1) with no observable differences in biological properties compared to the parental isolate was used in the paired-culture method as previously described (Wu et al., 2007, 2010; Jiang et al., 2015). This method was used to transfer AfPV1 from isolate LD-3-8 (donor) to the virus-free isolate LD-F1 (recipient) on CZ media (Czapek Agar) (Figure 4A). After 10 days of paired-culture, three mycelial agar plugs (marked “1,” “2,” and “3”; Figure 4A) from the colony of virus-free isolate LD-F1 were placed onto a fresh CZ plate containing 0.2 μg/ml pyrithiamine at 30°C and subcultured through five serial transfers onto pyrithiamine-amended media to allow only labeled isolates to grow. Finally, mycelial plugs from the new colonies were placed onto fresh PDA and CZ plates without pyrithiamine. AfPV1 was detected as described above. Total RNA of each culture was extracted using the TRIzol reagent (Invitrogen, United States), and was then used for the RT-PCR reaction with a random nonamer primer 5′-d (NNNNNNNNN)-3′. The specific primers (AfPV-det1: 5′-TCAAATACTACACGAAGGACGAAC-3′ and AfPV-det2: 5′-CATAGGCGGAGAAATCCAGAC-3′) were designed for specific detection of AfPV1.

### Growth Rate, Sporulation, and Murine Virulence of *A. flavus*

A square agar plug (5 mm wide) from the margin of each colony was inoculated onto the center of PDA and CZ plates (9 cm in diameter), and cultured at 30°C in the dark. The radial growth rate (RGR) was determined for each developing colony (five replicates for each fungal isolate) at 2 days intervals over a week period as follows: RGR (cm/d) = [(D 4-D 2)/2]/2, where D 4 and D 2 represent the diameter of 4 and 2 days old colonies in each plate, respectively.

After 6 days incubation in the dark at 30°C on PDA plates (9 cm in diameter), conidiospores of *A. flavus* were harvested in 2 mL saline buffer, and spore numbers for each colony were estimated using a haemocytometer; the data were transformed to log 10 before analyses. The length of spore chains were measured under microscope.

In order to assess *A. flavus* burden quantitatively, quantitative PCR (qPCR) was tested on total genomic DNA extracted from homogenized whole lungs of mice using a previously described method (Bhatti et al., 2011; Özkan and Coutts, 2015). For analysis of fungal burden in corticosteroid-treated female mice (Kunming mice, aged 6–8 weeks), immunosuppression was induced with subcutaneous injections of cyclophosphamide (200 mg/kg on days -3, -1, and +2). Spores were harvested from *A. flavus* cultured on PDA medium for 6 days at 30°C. On day 0 inocula containing about 1 × 10^7^ conidia were prepared from *A. flavus* isolates in 30 μl of saline buffer. Mice were anesthetized by injection of chloral hydrate and infected by intranasal instillation. In order to prevent bacterial infections, the drinking water was added 100 mg/L cephalosporin. *A. flavus* burden was assessed by qPCR amplification using the specific primers (Aftub-f:CTAGTGAAGTCTGAGTTGATTGTAT and Aftub-r:CCGGAGAGGGGACGACGA) to amplify *A. flavus*β-tubulin (M38265), and the specific primers (Muact-f:GAGACCTTCAACACCCCAGC and Muact-r: ATGTCACGCACGATTTCCC) to amplify the murine actin (NM_007393) as an internal control. Quantification of both gene targets was performed in triplicate, using ten independent infected mice, and saline-injected mice as control. qPCR assay was performed using SYBR green I (TAKARA, Dalian, China). The data of threshold cycle (Ct) were analyzed with unpaired *t*-tests using GraphPad Prism 5.01 (GraphPad Software, La Jolla, CA, United States). In order to confirm this model represent the virulence of the fungus *A. flavus*, microscopic and histological changes of mice lung tissues were observed after infection using a previously described method (Lan et al., 2018). The whole lungs of infected mice were putted in 10% paraformaldehyde, and then stained with Haema eosin (HE) for histologic examination. In addtion, lung tissues were also homogenized and diluted by sterile water, and the suspends were coated on PDA plus streptomycin and pyrithiamine at 30°C for *A. flavus* detection.

## Results

### Complete Sequence and Organization of AfPV1 Genome

**FIGURE 1:**
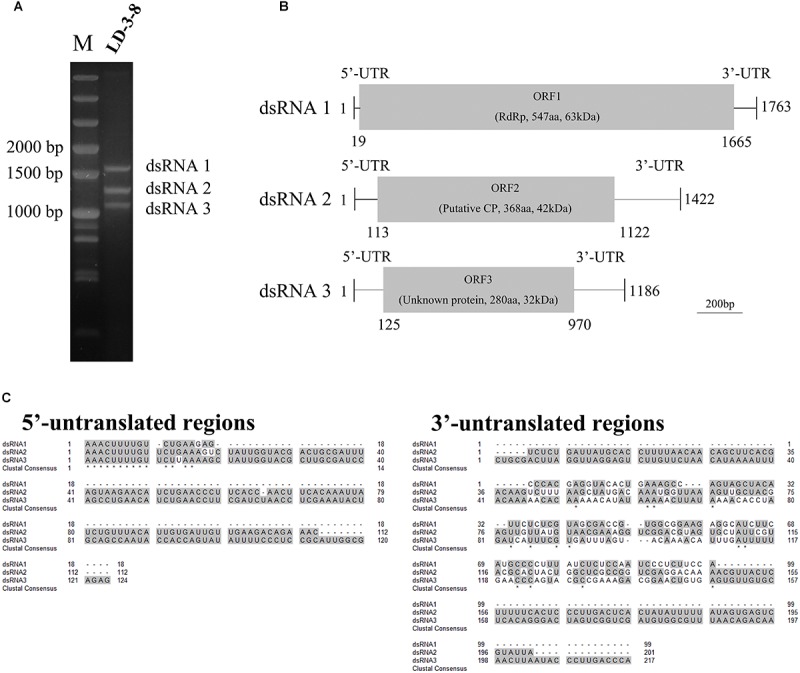
Genomic organization of AfPV1. **(A)** Agarose gel electrophoresis of the dsRNA segments extracted from *Aspergillus flavus* isolate LD-3-8. The molecular sizes are indicated on the left with numbers. **(B)** Genome organization of dsRNA1, dsRNA2, and dsRNA3 indicated by a graphical representation. The dsRNA1 is 1763 bp long and contains an ORF (designated as ORF1), which encodes a putative RdRp with a molecular mass of 63 kDa. The dsRNA2 is 1422 bp long and contains an ORF (designated as ORF2), which encodes a putative coat protein (CP) with a molecular mass of 42 kDa. The dsRNA3 is 1186 bp long and contains an ORF (designated as ORF3), which encodes a protein of unknown function with a molecular mass of 32 kDa. aa, amino acids. **(C)** Identity at the 5′-and 3′-UTR ends of the coding strands of the three genomic segments of AfPV1. Gray shading indicates identical nucleotides in the three segments.

Three segments (dsRNA1, dsRNA2, and dsRNA3) were detected in single spore isolate LD-3-8 (Figure 1A). The full-length cDNA sequences of those segments were obtained by assembling sequences from their respective overlapping cDNA clones. Results showed that dsRNA1 (GenBank Acc No. MK344768) is 1763 bp long with a GC content of 46.2%, dsRNA2 (GenBank Acc No. MK344769) is 1422 bp long with a GC content of 46.7%, and dsRNA3 (GenBank Acc No. MK344770) is 1186 bp long with a GC content of 45.4% (Figure 1B). Sequencing analysis indicated that dsRNA1 contains a single ORF from nucleotides (nt) 19 to 1665, which putatively encodes a protein with 547 amino acid (aa) residues and a molecular mass of 63 kDa (Figure 1B). The dsRNA2 encodes a single ORF encoding a putative 368-aa protein with a molecular mass of 42 kDa (Figure 1B), and dsRNA3 single ORF encodes a putative 280-aa protein with a molecular mass of 32 kDa (Figure 1B).

BlastP search indicated that the dsRNA1 product shares highly significant similarities with the RdRp of several viruses in the family *Partitiviridae*, including Botryosphaeria dothidea virus 1 (83% aa sequence identities, GenBank Acc No. AIE47694), *Colletotrichum acutatum* RNA virus 1 (76% aa sequence identities, GenBank Acc No. AGL42312), and *Valsa cypri* partitivirus (85% aa sequence identities, GenBank Acc No. AIS37548) (Table 1). No other significant similarity can be found between the putative protein encoded by dsRNA2 and any other CP sequences, except identities of 76 and 59% with the hypothetical proteins encoded by Botryosphaeria dothidea virus 1 and *C. acutatum* RNA virus 1 (Table 1). Moreover, no homologous sequences were found when dsRNA3 was queried in the GenBank database. The percent of amino acid identities are listed in Table 1, when dsRNA1 and dsRNA2 were queried in the GenBank database by BlastP.

**Table 1:** BlastP analysis of AfPV1 compared to the closest matching sequences in NCBI database, and the percent amino acid identities of putative protein encoded by dsRNA1 and dsRNA2.

Virus	RNA-dependent RNA polymerase (dsRNA1)		hypothetical protein (dsRNA2)	
	Identities	E-value	Identities	E-value
*Botryotinia fuckeliana* partitivirus 1	450/544 (83%)	0.0	281/370 (76%)	0.0
*Colletotrichum acutatum* RNA virus 1	409/539 (76%)	0.0	217/370 (59%)	4e-150
*Valsa cypri* partitivirus	375/440 (85%)	0.0	176/236 (75%)	2e-119
*Ustilaginoidea virens* partitivirus 3	268/513 (52%)	0.0	96/359 (27%)	2e-22
*Ustilaginoidea virens* partitivirus 2	264/526 (50%)	2e-174	107/361 (30%)	2e-25
*Ophiostoma* partitivirus 1	165/515 (32%)	1e-74	N	N
*Aspergillus ochraceous* virus	187/539 (35%)	8e-74	N	N
*Sodiomyces alkalinus* partitivirus 2	167/494 (34%)	4e-73	N	N

*N means no significant similarity was found via NCBI BlastP analysis. Identity means the identical or similar amino acid residues/total length of the amino acid residues used in sequence comparison analysis. The GenBank accession numbers of the selected viruses are shown in Supplementary Table S1*.

The 5′-untranslated regions (UTRs) of the plus strands of dsRNA1, dsRNA2 and dsRNA3 are 18, 112, and 124 bp long, respectively, and show conserved nucleotides at the 5′ terminus (AAACUUUUGU) (Figure 1C). A similar motif (AAACUUUUG/AU/G) is present in the genomes of Botryosphaeria dothidea virus 1. The 3′-UTRs differ in length, and there is little sequence conservation (Figure 1C). This is consistent with observations for multi-component RNA viruses, where the 5′ terminal sequences are essential for recognition by the virus RdRp during viral RNA replication. Such conserved sequences near the 5′ termini of partitiviruses are thought to be involved in RdRp recognition for RNA packaging and/or replication (Nibert et al., 2014).

**FIGURE 2:**
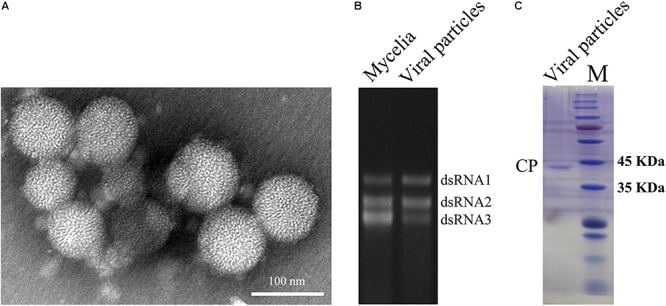
Virus particles of AfPV1. **(A)** TEM observation of the virus particles from *A. flavus* isolate LD-3-8. **(B)** Agarose gel electrophoresed analysis of dsRNA profiles from mycelia and viral particles. **(C)** SDS-PAGE analysis of the purified virus particles showing the 42 kDa protein components. The molecular weight of the protein bands was estimated on the right with numbers.

To obtain dsRNA segregation patterns in isolates, we conducted a transmission test of the dsRNAs to asexual conidia spores and other *A. flavus* isolates. In our study, dsRNA1, dsRNA2, and dsRNA3 could not be detected separately and they always appear to coexist in *A. flavus*. Moreover, the three segments appear to coexist in purified VLPs (Figure 2B). These results should suggest that the dsRNAs (dsRNA1, dsRNA2, and dsRNA3) belong to the same virus, and this virus is to be assigned to the family *Partitiviridae.* So we named this virus as AfPV1.

### Observation and PMF-MS Analyses of Virus Particles

Whether virus particles really exist in the virus preparations, the preparations were subjected to TEM. Spherical VLPs of about 40 nm in diameter were detected under TEM after purification (Figure 2A). Three dsRNA segments released from purified VLPs were similar in size to those isolated directly from mycelia of isolate LD-3-8 (Figure 2B). The purified VLPs were also examined by SDS-PAGE and Coomassie brilliant blue staining, and a single major band with molecular weight of 42 kDa possibly represents the CP (Figure 2C). The band was extracted from a gel and subjected to PMF-MS analysis. Five peptide fragments with ion scores higher than 31 (identity or extensive homology, *p* < 0.05), matched to the deduced amino acid sequence of the protein encoded by dsRNA2 (Supplementary Table S2). To avoid missing any proteins during virion preparation, the purified virus particles suspension was also directly subjected to PMF-MS analysis. Seventeen peptide fragments with ion scores higher than 0 (identity or extensive homology, *p* < 0.05), matched to the deduced amino acid sequence of the protein encoded by dsRNA2 (Supplementary Table S3). Both PMF-MS results did not match to the deduced amino acid sequence of the protein encoded by dsRNA1 or dsRNA3. The results unequivocally indicated that the CP of the AfPV1 was encoded by dsRNA2, which is similar to the finding for *C. acutatum* RNA virus 1 (Zhong et al., 2014a).

### Phylogenetic Analysis of AfPV1

**FIGURE 3:**
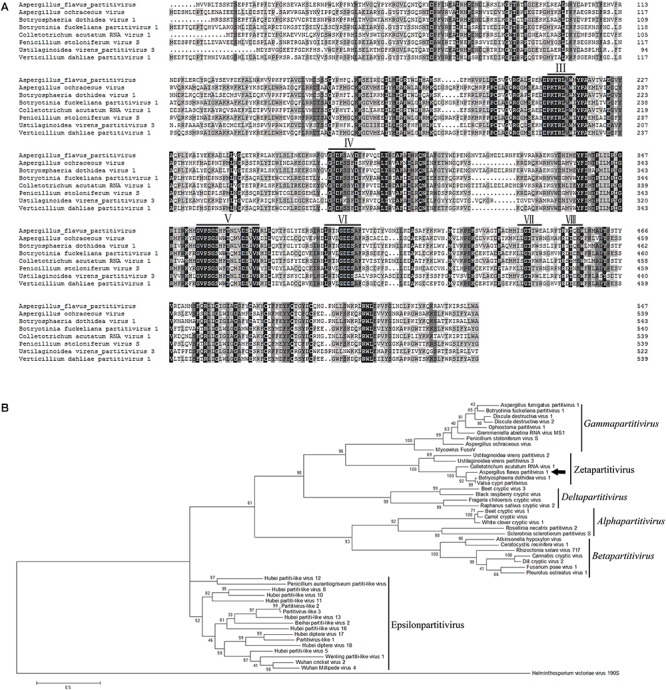
Phylogenetic analysis of AfPV1. **(A)** Amino acid sequence alignment of the core RdRp motifs of AfPV1 and other selected mycoviruses in the family *Partitiviridae*. Similar amino acid residues are shaded in gray. The conserved motifs of RdRp in dsRNA viruses are indicated by numbers (Bruenn, 1993). **(B)** Phylogenetic analysis of the RdRp sequences of AfPV1 and other selected dsRNA mycoviruses in family of *Partitiviridae*. The phylogenic tree was generated based on the Maximum Likelihood method with a 1000-replicate bootstrap search using the program MEGA 6.06, and the bootstrap values are indicated at the branch points. Moreover, the tree was rooted with the RdRp of Helminthosporium victoriae virus 190S, a member of the genus *Totivirus* in the family *Totiviridae*. The arrow indicates the position of AfPV1. The GenBank accession numbers of the selected viruses are shown in Supplementary Table S1.

Multiple alignments of RdRp amino acid sequences of the representative viruses in the family *Partitiviridae* were performed and revealed that the RdRp domain of AfPV1 contains six conserved motifs in the putative RdRps of mycoviruses (Bruenn, 1993; Figure 3A). To analyze the phylogenetic position of AfPV1, a phylogenetic tree was constructed using deduced amino acid sequences of the RdRp of AfPV1 and other selected dsRNA viruses from the family *Partitiviridae*. These viruses revealed four recognized genera (*Alphapartitivirus,* *Betapartitivirus,* *Gammapartitivirus,* and *Deltapartitivirus*) and a new genus (Epsilonpartitivirus) in family of *Partitiviridae* (Nerva et al., 2017; Vainio et al., 2018). This analysis shows that AfPV1 and relative viruses (Botryosphaeria dothidea virus 1, *C. acutatum* RNA virus 1, *V. cypri* partitivirus, *U. virens* partitivirus 2, and *U. virens* partitivirus 3) were found in a unclassified cluster, and this cluster grouped clearly outside of the five genera in *Partitiviridae* family (Figure 3B).

### Horizontal Transmission and the Effect of AfPV1 in *A. flavus*

**FIGURE 4:**
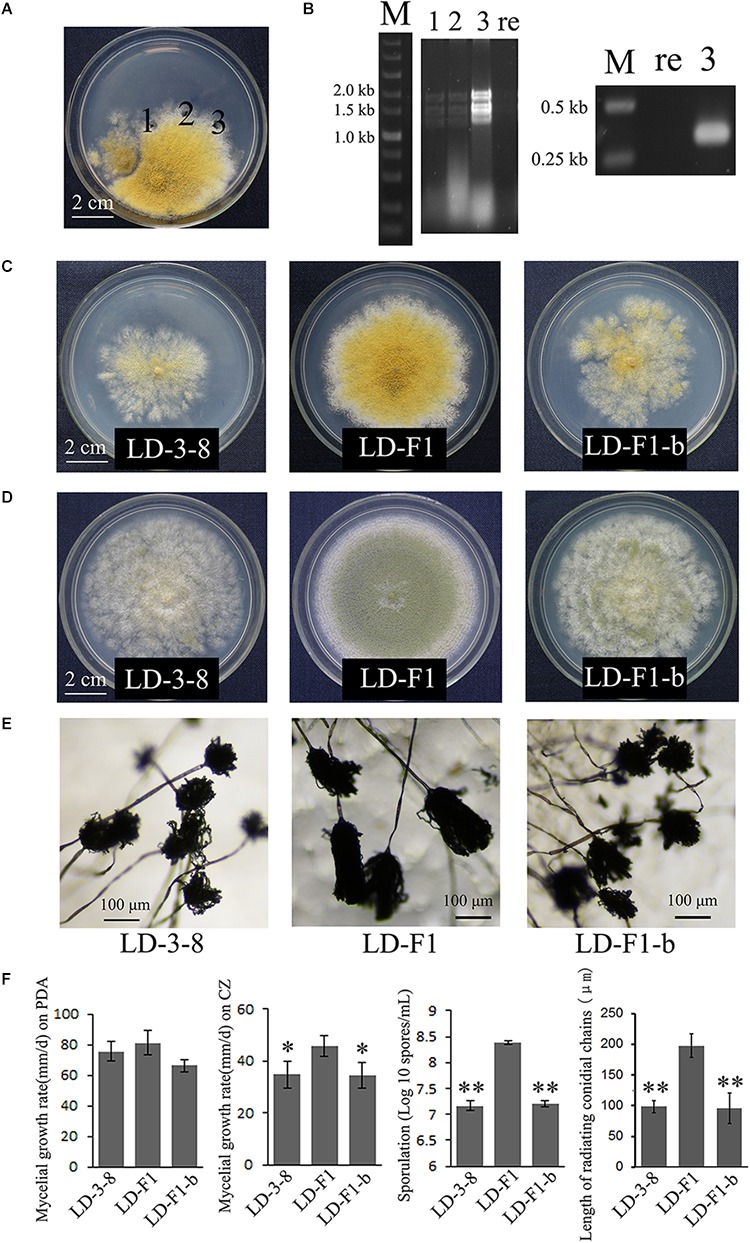
Biological effects of AfPV1 in *A. flavus.* **(A)** Paired-cultures between the donor isolate LD-3-8 (left) and the virus-free recipient isolate LD-F1 (right). Derivative isolates were obtained from the mycelia agar plugs marked “1,” “2,” and “3”. **(B)** Agarose gel electrophoresis of dsRNA extracted from derivative isolates (left) and RT-PCR detection for AfPV1 (right). Lane re is recipient isolate LD-F1; lane 3 is derivative isolate LD-F1-b; lane 1, 2, and 3 in panels **B** correspond to panels **A**. Colony morphology of isolates LD-3-8, LD-F1 and LD-F1-b on CZ **(C)**, and PDA **(D)** for 6 days. **(E)** Conidial heads of isolates LD-3-8, LD-F1, and LD-F1-b.hfill **(F)** Comparison of the growth rate, sporulation and length of radiating chains of conidia among isolates LD-3-8, LD-F1, and LD-F1-b. Isolate LD-3-8 was infected with AfPV1, virus-free isolate LD-F1 was obtained from isolate LD-3-8 by single asexual spore isolates, and then was labeled with a pyrithiamine resistance (*ptr*) gene, and isolate LD-F1-b was one of the derivative isolates, which were obtained by transferring AfPV1 from isolate LD-3-8 (donor) to the virus-free isolate LD-F1 (recipient). “^∗^” represented significantly different at the *P* < 0.05 level of confidence and “^∗∗^” represented significantly different at the *P* < 0.01 level of confidence according to Turkey’s multiple comparison test.)

Using horizontal transmission methods, the virus-free strain LD-F1 was successfully transfected with AfPV1. Three derivative isolates were obtained from a recipient colony of LD-F1. The mycelia agar plugs were marked “1,” “2,” and “3” (Figure 4A). After subculturing on a CZ plate (0.2 μg/ml pyrithiamine) for 5 generations, the derivative isolates were used for both dsRNA extraction and RT-PCR amplification (Figure 4B).

Compared to virus-free isolate LD-F1, the newly AfPV1-infected isolate LD-F1-b displayed debilitation symptoms, including abnormal colonial morphology, slow growth on CZ, and poor sporulation, and that is similar to AfPV1-infected isolate LD-3-8 (Figure 4C–F). Moreover, we found that spore chains of these AfPV1-infected isolates (LD-3-8 and LD-F1-b) are shorter, indicating a possible lower sporulation potential (Figure 4E,F). Although the AfPV1-infected isolates have caused significantly abnormal colonial morphology (Figure 4C,D), the growth rates are not significantly different between AfPV1-infected isolates and virus-free isolates on PDA (Figure 4F).

**FIGURE 5:**
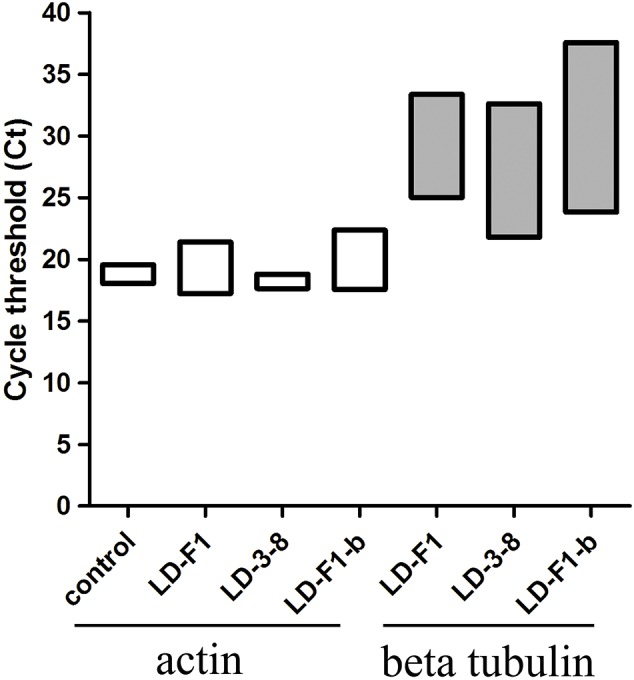
Quantitative PCR (qPCR) showing fungal burden in the mice model. Cycle of threshold (Ct) values are measured for *A. flavus*β-tubulin and mice actin genes from total genomic DNA, extracted from homogenized lung tissue, following infection with isolates LD-F1, LD-3-8, and LD-F1-b. While expression of mice actin gene was measured in the saline-injected mice, no *A. flavus*β-tubulin gene expression was observed.

In the murine virulence assays, the lungs tissue of the *A. flavus* (virus-free isolate LD-F1 and AfPV1-infected isolate LD-F1-b) infected mice appeared large numbers of bleeding area compared to saline-injected mice, but no significant differences between LD-F1 and LD-F1-b infected mice (Supplementary Figure S1). Afer cultured on PDA, *A. flavus* was detected in mice lungs tissue of *A. flavus* (isolate LD-F1 and LD-F1-b) infection, but was not detected in saline-injected mice (Supplementary Figure S1). qPCR was performed using the accumulation levels of the *A. flavus*β-tubulin gene as an indication of fungal growth and burden in mice lungs inoculated with virus-free or AfPV1-infected isogenic lines of *A. flavus*. The mice actin gene that was used as an internal control, remained at the same level in all of the qPCR experiments, and there was no β-tubulin gene expression in all saline-injected mice (Figure 5). The statistical significance of variances between fungal burdens were calculated using a non-parametric *t*-test. The accumulation levels of β-tubulin were not significantly different between the virus-free and AfPV1-infected isolates (*P* = 0.8; Figure 5).

## Discussion

*Aspergillus flavus* is an important fungal pathogen that not only induces diseases in human, animals and plants, but also produces aflatoxins with strong carcinogenic and mutagenic effects (Amaike and Keller, 2011). Nevertheless, mycoviruses are rarely reported in *A. flavus*, only and isolate NRRL 5565 and NRRL 5940 was reported to harbor dsRNA elements (Kotta-Loizou and Coutts, 2017). In this study, we isolated a novel mycovirus (AfPV1) with isometric particles 40 nm in diameter from an abnormal phenotype isolate (LD-3-8) of *A. flavus*. Hypovirulence-associated mycoviruses have shown the significant potential for biological control agents, but the potential of mycoviruses to combat fungal infection in animals and humans is still unknown (Van De Sande et al., 2010; Bhatti et al., 2011; Özkan and Coutts, 2015). Mycoviruses that confer hypovirulence are generally characterized by a reduction in conidiation, pigmentation and growth rate (Son et al., 2015). So we were encouraged by the fact that AfPV1 caused obvious effects on *A. flavus,* such as abnormal colonial morphology, slow growth on CZ, and poor sporulation (Figure 4). Nevertheless, we could not detect any significant effect of AfPV1 on its host virulence (Figure 5). Histological and microscopic observition of mice lung indicated that the murine model was suitable for testing virulence of *A. flavus* (Supplementary Figure S1). The pathogenicity of aspergilli are tested in both murine and moth infection models (Ford and Friedman, 1967; Bhatti et al., 2011; Özkan and Coutts, 2015; Negri et al., 2017). Both systems have advantages and limitations (Desalermos et al., 2012; Arvanitis et al., 2013). Our tests on the effects of AfPV1 on virulence in *A. flavus* confirmed the utility and reliability of murine model.

Based on nucleotide sequence analysis, AfPV1 is unambiguously identified as a new member of the family *Partitiviridae.* Most partitiviruses generally possess two essential dsRNA genome segments (1.3–2.5 kbp in length), the larger one encodes RNA-dependent RNA polymerase (RdRp) and the smaller one encodes CP (Nibert et al., 2014). Moreover, the presence of one or more additional dsRNA segments is common in *Partitiviridae* family (Nibert et al., 2014). The genome of AfPV1 contains three segments (dsRNA1 with 1.7 kbp, dsRNA2 with 1.4 kbp, and dsRNA3 with 1.2 kbp), and the 5′-untranslated regions (UTRs) of dsRNA1, dsRNA2 and dsRNA3 show high sequence identity with each other, which is a feature of partitiviruses (Nibert et al., 2014).

DsRNA1 encodes the RdRp of AfPV1 and dsRNA2 is considered to encode the CP of AfPV1 which is similar to the finding for *C. acutatum* RNA virus 1 (Zhong et al., 2014a). Moreover, the CP of AfPV1 shows similarity to hypothetical protein encoded by Botryosphaeria dothidea virus 1, *C. acutatum* RNA virus 1, *V. cypri* partitivirus, *U. virens* partitivirus 2, and *U. virens* partitivirus 3 (Table 1), but do not have any similarity to partitiviruses in five genera (*Alphapartitivirus,* *Betapartitivirus,* *Gammapartitivirus*, *Deltapartitivirus,* and Epsilonpartitivirus). These results indicates the CPs of the viruses in this group may be new CP category in family *Partitiviridae.* AfPV1 and Botryosphaeria dothidea virus 1 contains three segments in each genome, but *C. acutatum* RNA virus 1, *U. virens* partitivirus 2 and *U. virens* partitivirus 3 possesses two segments in each genome, that is the difference in this group (Zhong et al., 2014a,b,c). Recently, new viral sequences associated to invertebrates and fungi were defined as a new genus “Epsilonpartitivirus” in family *Partitiviridae* (Shi et al., 2016; Nerva et al., 2017). The phylogenetic analysis indicates that the AfPV1, Botryosphaeria dothidea virus 1, *C. acutatum* RNA virus 1, *V. cypri* partitivirus, *U. virens* partitivirus 2 and *U. virens* partitivirus 3 are clearly grouped in an unclassified taxon, which is outside the five existing genera in *Partitiviridae* family (Figure 3B). Taking these aspects into consideration, we suggest that AfPV1 and relative viruses may group as a new genus named “Zetapartitivirus” according to the nomenclature of genera in *Partitiviridae* family.

Members of the family *Partitiviridae* are usually considered to have latent effect infections of their hosts, such as resistance to salinity, regulation of mycotoxin production or biocontrol potential (Nibert et al., 2014; Nerva et al., 2017, 2018; Chun et al., 2018). However, only few partitiviruses are likely responsible for inducing abnormal phenotypes or virulence reduction in their hosts. Sclerotinia sclerotiorum partitivirus 1 (SsPV1) and Rhizoctonia solani partitivirus 2 (RsPV2) conferred hypovirulence on its natural plant-pathogenic fungal host; Flammulina velutipes browning virus (FvBV) is known to be associated with the cap color of the fruiting bodies of *Flammulina velutipes*; Aspergillus fumigatus partitivirus 1 (AfuPV1) caused abnormal colony phenotypes, slow growth, and production of light pigmentation on its host *A. fumigatus* (Magae and Sunagawa, 2010; Bhatti et al., 2011; Xiao et al., 2014; Zheng et al., 2014). In our study, the AfPV1-infected isolates displayed debilitation symptoms, including abnormal colonial morphology, slow growth on CZ, poor sporulation, and short spore chains. However, there are no significant impact of AfPV1 on the virulence of its host, which is similar to AfuPV1 (Bhatti et al., 2011; Krishnan et al., 2009).

In conclusion, we have isolated and characterized a new partitivirus with 3 genome segments and provided convincing evidence that dsRNA2 encodes a CP, whereas the function of the ORF encoded by dsRNA3 remains unknown. Phylogenetic analysis suggest that AfPV1 belongs to a new genus inside the *Partitiviridae* family for which we propose the name Zetapartitivirus. Horizontal transmission analysis revealed that AfPV1 infection causes debilitation symptoms but no significant alterations to murine pathogenicity of *A. flavus*.

## Author Contributions

YJ designed and analyzed the data. JW collected and analyzed the data. BY purified the virus particles. JZ analyzed the mice aspergillosis model. QW and WY gave some advises for the work.

## Conflict of Interest Statement

The authors declare that the research was conducted in the absence of any commercial or financial relationships that could be construed as a potential conflict of interest.

## Supplementary Material

FIGURE S1 Histological and microscopic observition of mice lung of control and *A. flavus* (isolate LD-F1 and LD-F1-b) infection. **(A)** Histological changes of mice lungs. **(B)**. Hematoxylin eosin staining analysis of mice lungs. **(C)** Culture detection of *A. flavus* on PDA. Control was saline-injected mice. Bars = 200 μm.

TABLE S1 NCBI protein database accession numbers of the virus used for phylogenetic analysis.

TABLE S2 The matrix-assisted laser desorption/ionization-time of flight mass spectrometry (MALDI-TOF-MS) analysis of protein bands on the gel. Ions score is -10 log(*P*), where *P* is the probability that the observed match is a random event. Protein scores are derived from ions scores as a non-probabilistic basis for ranking protein hits. Individual ions scores >31 indicate identity or extensive homology (*p* < 0.05). The overall protein score is 268 ppm means peptide mass tolerance.

TABLE S3 The liquid chromatography-tandem mass spectrometry analysis of purified virus particles. Ions score is -10 log(*P*), where *P* is the probability that the observed match is a random event. Protein scores are derived from ions scores as a non-probabilistic basis for ranking protein families. Individual ions scores >0 indicate identity or extensive homology (*p* < 0.05). The overall protein score is 1245 ppm means peptide mass tolerance.
