# Supplementary figures and images for: Molecular Characterization of a Debilitation-Associated Partitivirus Infecting the Pathogenic Fungus Aspergillus flavus
Source: Front Microbiol. 2019 Mar 28;10:626. doi: 10.3389/fmicb.2019.00626 (PMC6447663; doi:10.3389/fmicb.2019.00626)

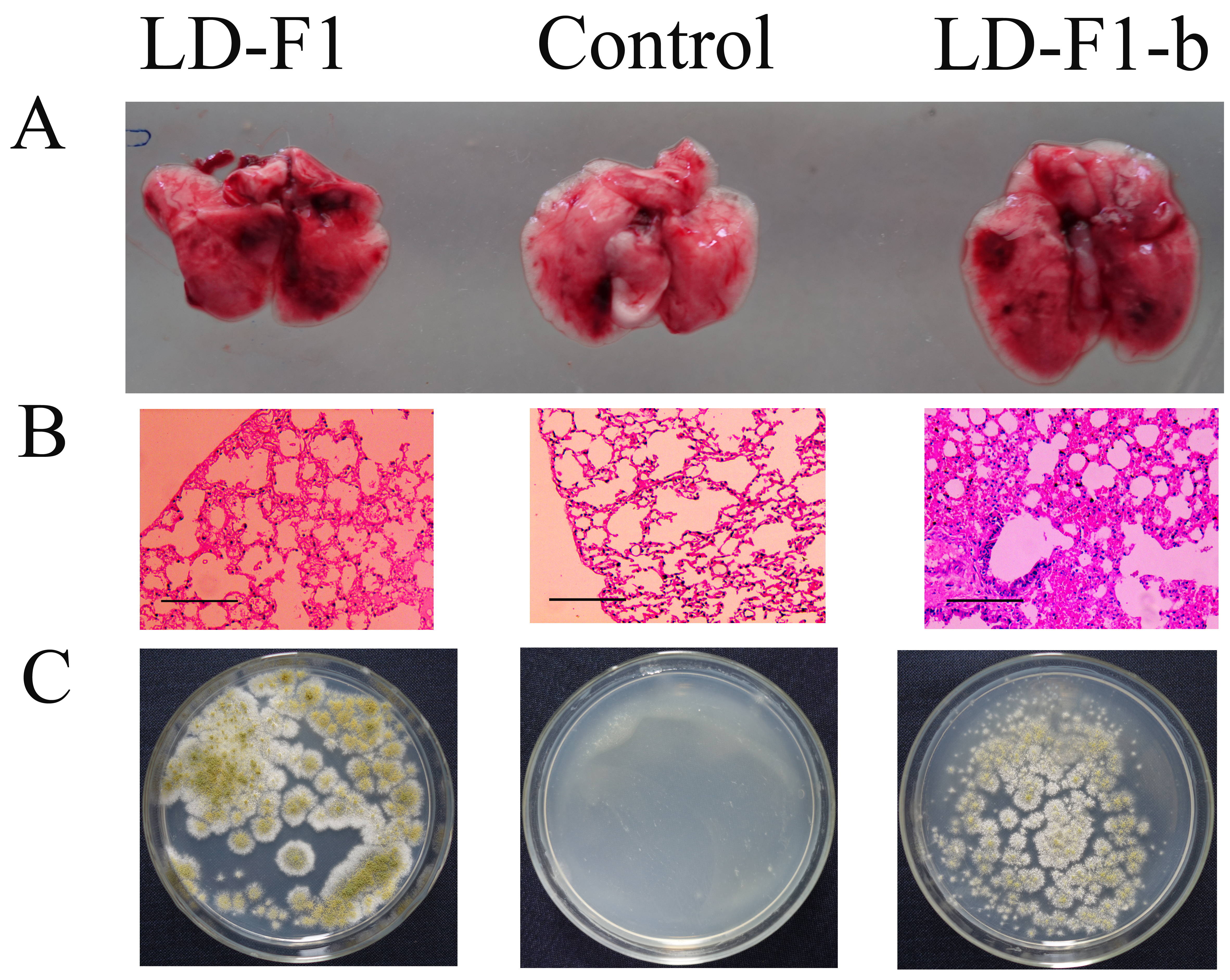

Supplement: FIGURE S1 — Histological and microscopic observition of mice lung of control and A. flavus (isolate LD-F1 and LD-F1-b) infection. (A) Histological changes of mice lungs. (B). Hematoxylin eosin staining analysis of mice lungs. (C) Culture detection of A. flavus on PDA. Control was saline-injected mice. Bars = 200 μm. [file Image_1.JPEG]
