# Supplementary material for: Molecular Characterization of a Debilitation-Associated Partitivirus Infecting the Pathogenic Fungus Aspergillus flavus
Source: Front Microbiol. 2019 Mar 28;10:626. doi: 10.3389/fmicb.2019.00626 (PMC6447663; doi:10.3389/fmicb.2019.00626)
Supplement: TABLE S1 — NCBI protein database accession numbers of the virus used for phylogenetic analysis. [file Table_1.DOC]

Table S1 NCBI protein database accession numbers of the virus used for phylogenetic analysis.

| Virus | Accession number | |
| --- | --- | --- |
| Aspergillus fumigatus partitivirus 1 | | CAY25801.2 |
| Aspergillus ochraceous virus | | ABV30675.1 |
| Atkinsonella hypoxylon virus | | AAA61829.1 |
| Beet cryptic virus 1 | | ACA81389.1 |
| Beet cryptic virus 3 | | AAB27624.1 |
| Beihai partiti-like virus 2 | | YP_009333350.1 |
| Black raspberry cryptic virus | | ABU55400.1 |
| Botryosphaeria dothidea virus 1 | | AIE47694.1 |
| Botryotinia fuckeliana partitivirus 1 | | CAM33266.1 |
| Cannabis cryptic virus | | AET80948.1 |
| Carrot cryptic virus | | ACL93278.1 |
| Ceratocystis resinifera virus 1 | | AAU26069.1 |
| Colletotrichum acutatum RNA virus 1 | | AGL42312.1 |
| Dill cryptic virus 2 | | AGJ83771.1 |
| Discula destructiva virus 1 | | AAG59816.1 |
| Discula destructiva virus 2 | | AAK59379.1 |
| Fragaria chiloensis cryptic virus | | AAZ06131.2 |
| Fusarium poae virus 1 | | AAC98734.1 |
| Helminthosporium victoriae virus 190S | | NP_619670.2 |
| Hubei partiti-like virus 8 | | APG78307.1 |
| Hubei partiti-like virus 10 | | APG78227.1 |
| Hubei partiti-like virus 11 | | YP 009329875.1 |
| Hubei partiti-like virus 12 | | APG78257.1 |
| Hubei partiti-like virus 13 | | APG78275.1 |
| Hubei partiti-like virus 16 | | APG78316.1 |
| Hubei diptera virus 17 | | YP_009337870.1 |
| Hubei diptera virus 18 | | YP_009329892.1 |
| Mycovirus FusoV | | BAA09520.1 |
| Ophiostoma partitivirus 1 | | YP_009508238.1 |
| Partitivirus-like 1 | | AOR51388.1 |
| Partitivirus-like 2 | | AOR51389.1 |
| Partitivirus-like 3 | | AOR51390.1 |
| Penicillium aurantiogriseum partiti-like virus | | ALO50133.1 |
| Penicillium stoloniferum virus S | | AAN86834.2 |
| Pleurotus ostreatus virus 1 | | AAT07072.1 |
| Raphanus sativus cryptic virus 2 | | ABB04855.1 |
| Rhizoctonia solani virus 717 | | AAF22160.1 |
| Rosellinia necatrix partitivirus 2 | | BAM78602.1 |
| Sclerotinia sclerotiorum partitivirus S | | ACT55329.1 |
| Ustilaginoidea virens partitivirus 2 | | YP_008327313.1 |
| Ustilaginoidea virens partitivirus 3 | | AGJ03719.1 |
| Valsa cypri partitivirus | | AIS37548.1 |
| White clover cryptic virus 1 | | AAU14888.1 |
