# Supplementary material for: Molecular Characterization of a Debilitation-Associated Partitivirus Infecting the Pathogenic Fungus Aspergillus flavus
Source: Front Microbiol. 2019 Mar 28;10:626. doi: 10.3389/fmicb.2019.00626 (PMC6447663; doi:10.3389/fmicb.2019.00626)
Supplement: TABLE S2 — The matrix-assisted laser desorption/ionization-time of flight mass spectrometry (MALDI-TOF-MS) analysis of protein bands on the gel. Ions score is -10 log(P), where P is the probability that the observed match is a random event. Protein scores are derived from ions scores as a non-probabilistic basis for ranking protein hits. Individual ions scores >31 indicate identity or extensive homology (p < 0.05). The overall protein score is 268 ppm means peptide mass tolerance. [file Table_2.DOCX]

Table S2. The matrix-assisted laser desorption/ionization-time of flight mass spectrometry (MALDI-TOF-MS) analysis of protein bands on the gel.

| Amino acid position | Calculated mass | Expected mass | ppm | Amino acid sequence | Ion score |
| --- | --- | --- | --- | --- | --- |
| 145-165 | 2532.0942 | 2532.0010 | -36.8 | DLDWNDENPTVHDTFNGWTTR | 43 |
| 166-183 | 2047.0222 | 2046.9328 | -43.7 | LNLNSTGEIQVDYTEPVR | 160 |
| 244-252 | 1106.5794 | 1106.5270 | -47.3 | IEAFEMVLR | 54 |
| 253-263 | 1303.6309 | 1303.5712 | -45.8 | QAFPNDVPNFR | 49 |
| 285-301 | 2002.0380 | 2001.9488 | -44.5 | NVVEELFINMAIVQMPR | 71 |

Note: Ions score is -10 log(*P*), where *P* is the probability that the observed match is a random event. Protein scores are derived from ions scores as a non-probabilistic basis for ranking protein hits.Individual ions scores > 31 indicate identity or extensive homology (p<0.05). The overall protein score is 268. ppm means peptide mass tolerance.
