# Supplementary material for: Molecular Characterization of a Debilitation-Associated Partitivirus Infecting the Pathogenic Fungus Aspergillus flavus
Source: Front Microbiol. 2019 Mar 28;10:626. doi: 10.3389/fmicb.2019.00626 (PMC6447663; doi:10.3389/fmicb.2019.00626)
Supplement: TABLE S3 — The liquid chromatography-tandem mass spectrometry analysis of purified virus particles. Ions score is -10 log(P), where P is the probability that the observed match is a random event. Protein scores are derived from ions scores as a non-probabilistic basis for ranking protein families. Individual ions scores >0 indicate identity or extensive homology (p < 0.05). The overall protein score is 1245 ppm means peptide mass tolerance. [file Table_3.DOCX]

Table S3. The liquid chromatography-tandem mass spectrometry analysis of purified virus particles.

| Amino acid position | Calculated mass | Expected mass | ppm | Amino acid sequence | Ion score |
| --- | --- | --- | --- | --- | --- |
| 2-19 | 2064.0173 | 2064.0072 | -5 | APEFKPGCFEPVITVSMR | 41 |
| 26-43 | 1983.8785 | 1983.8725 | -3 | AFHGDSQGLFDGCLDAFK | 97 |
| 61-68 | 793.4004 | 793.3960 | -6 | AGMTSISK | 47 |
| 69-77 | 1152.5750 | 1152.5790 | 3 | VMAQYLWAR | 1 |
| 78-91 | 1645.7961 | 1645.7874 | -5 | QLSNQLAHFGFNDR | 83 |
| 109-122 | 1759.7899 | 1759.7893 | -5 | LFNCYGHVEHEEVK | 69 |
| 145-165 | 2532.0942 | 2532.0826 | -5 | DLDWNDENPTVHDTFNGWTTR | 69 |
| 166-183 | 2047.0222 | 2047.0135 | -4 | LNLNSTGEIQVDYTEPVR | 102 |
| 189-203 | 1724.8893 | 1723.8869 | -1 | LVAHINALDVDPEYR | 67 |
| 209-217 | 1031.5247 | 1031.5205 | -4 | NIIDVSTDR | 48 |
| 209-224 | 1874.9520 | 1987.9449 | -4 | NIIDVSTDRDLNTMLR | 47 |
| 218-224 | 861.4378 | 861.4330 | -6 | DLNTMLR | 28 |
| 228-238 | 1218.6469 | 1218.6433 | -3 | NQPNIGNLPPR | 28 |
| 244-252 | 1106.5794 | 1106.5744 | -4 | IEAFEMVLR | 64 |
| 253-263 | 1303.6309 | 1303.6245 | -5 | QAFPNDVPNFR | 34 |
| 271-278 | 936.5215 | 936.5176 | -4 | VMNPVIHK | 39 |
| 347-357 | 1129.5727 | 1129.5672 | -5 | ASPEQVGSSLR | 30 |

Note: Ions score is −10 log(*P*), where *P* is the probability that the observed match is a random event. Protein scores are derived from ions scores as a non-probabilistic basis for ranking protein families. Individual ions scores > 0 indicate identity or extensive homology (p<0.05). The overall protein score is 1245. ppm means peptide mass tolerance.
